# Microbial Diversity and Evidence of Novel Homoacetogens in the Gut of Both Geriatric and Adult Giant Pandas (*Ailuropoda melanoleuca*)

**DOI:** 10.1371/journal.pone.0079902

**Published:** 2014-01-24

**Authors:** Hein Min Tun, Nathalie France Mauroo, Chan San Yuen, John Chi Wang Ho, Mabel Ting Wong, Frederick Chi-Ching Leung

**Affiliations:** 1 School of Biological Sciences, The University of Hong Kong, Hong Kong, Hong Kong SAR; 2 Department of Pathology, The University of Hong Kong, Hong Kong, Hong Kong SAR; 3 Clinical Laboratory, Veterinary Center, Ocean Park Corporation, Hong Kong, Hong Kong SAR; 4 Hong Kong Wildlife Health Foundation, Hong Kong, Hong Kong SAR; 5 Bioinformatics Center, Nanjing Agricultural University, Nanjing, China; Naval Research Laboratory, United States of America

## Abstract

Recent studies have described the bacterial community residing in the guts of giant pandas, together with the presence of lignocellulolytic enzymes. However, a more comprehensive understanding of the intestinal microbial composition and its functional capacity in giant pandas remains a major goal. Here, we conducted a comparison of bacterial, fungal and homoacetogenic microbial communities from fecal samples taken from two geriatric and two adult captive giant pandas. 16S rDNA amplicon pyrosequencing revealed that Firmicutes and Proteobacteria are the most abundant microbiota in both geriatric and adult giant pandas. However, members of phylum Actinobacteria found in adult giant pandas were absent in their geriatric counterparts. Similarly, ITS1 amplicon pyrosequencing identified developmental changes in the most abundant fungal classes from Sordariomycetes in adult pandas to Saccharomycetes in geriatric pandas. Geriatric pandas exhibited significantly higher abundance of a potential probiotic fungus (*Candida tropicalis*) as compared to adult pandas, indicating their importance in the normal digestive physiology of aged pandas. Our study also reported the presence of a lignocellulolytic white-rot fungus, *Perenniporia medulla-panis*, and the evidence of novel homoacetogens residing in the guts of giant pandas.

## Introduction

The giant panda (*Ailuropoda melanoleuca*) is an endangered species endemic to China, with a population of less than 2,500 animals in the wild, and ∼200 in zoological institutions and breeding centers around the world [1], [2]. Low fecundity, low nutritional intake, and loss of habitats due to human activities and natural disasters are the predominant causes of the decreasing numbers of giant pandas [1]. As a member of the bear family (Ursidae), the giant panda possesses a gastrointestinal tract (GIT) typical of carnivores, yet intriguingly subscribes to an herbivorous diet consisting predominantly of bamboo. In captivity, the giant pandas devote around 25% of their daily time on feeding activities, consuming up to 14 kg of bamboo [3] and other non-bamboo supplementary foods such as fruits and high-fiber biscuits. Although pandas ingest highly fibrous diets, only 17% of the consumed dry matter is digested in their GITs [4], [5]. As revealed previously, giant pandas lack the putative genes encoding for enzymes degrading lignocelluloses, suggesting that microbial degradation plays a significant role in bamboo digestion [2].

Recently, several groups have attempted to characterize the microbial populations in the guts of giant pandas using both culture-dependent and -independent methods [6]–[8]. Recent advancements in sequencing technology have assisted in understanding the microbial role in lignocellulose degradation [9]. Additionally, the presence of a lignin-degrading related enzyme (laccase) in the giant panda fecal microbiome has been recently reported by metagenomic library screening [10]. However, the significance of these potential lignocellulose degraders remains dubious due to the selective dietary preferences exhibited by individual pandas [5], [6], and each individual diet would contribute differently towards the host GIT microflora composition [6], [11], [12].

Besides the lignocellulolytic bacterial community, the homoacetogenic bacterial (acetogens) and fungal communities are of further interest in the understanding of the microbial digestion and energy metabolism of giant pandas. The homoacetogens are a group of obligate anaerobes that employ the Wood-Ljungdahl pathway to synthesize acetate from CO_2_ [13]. In terms of prevalence, the homoacetogens were identified in ruminants and other non-ruminant hosts [14]–[18], but remain uncharacterized in giant pandas. Fungi, on the other hand, are ubiquitous components of a variety of natural ecosystems [19]–[22] and interact with other resident microbes to form complex ecosystem structures and functions [23]. To our understanding, no study has explored fungal diversity in the giant panda GIT.

Regarding the health of giant pandas, gastrointestinal diseases are the most common causes of mortality in both captive and wild giant pandas. [24]. In terms of longevity, captive giant pandas generally have a lifespan of almost 30 years (equivalent to 120 human years) [25]; since their reproduction generally ends after the age of 20, individuals beyond this age are considered to be “geriatric.” Although gastrointestinal disorders have been recognized predominantly in aged pandas [24], little information is available regarding geriatric pandas due to their considerably lower population numbers. In recent years, rapid improvements in husbandry and medical management have increased the number of geriatric pandas in zoological institutions [24]. As a path to an efficient conservation scheme of giant pandas, a comprehensive knowledge of their developmental changes in GIT microflora is crucial. While studies in human showed that the composition of intestinal microbiotas among geriatric individuals varies greatly [26], [27], and differs from that of young adults [27], [28]. Such information in geriatric and adult pandas remains unknown.

Thus, the purpose of this study was to determine both bacterial and fungal diversities in the guts of giant pandas by utilizing pyrotag sequencing, additional effort was also devoted to examine the homoacetogenic diversity due to their proposed capability to synthesize acetate from CO_2_ and H_2_. To this end, we have sampled two geriatric and two adult pandas living under captivity, allowing the first insight towards the microbial compositions between two different age groups.

## Results

### Bacterial diversity in the guts of giant pandas

**Figure 1:**
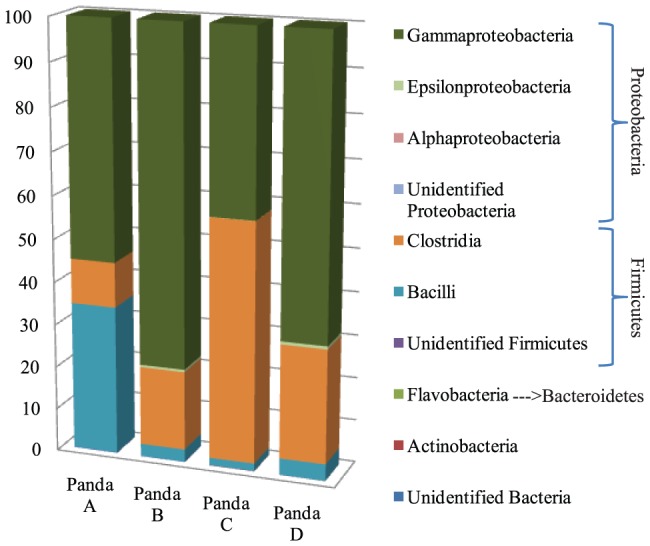
Bacterial diversity among four giant pandas. The bar graph represents the relative distribution of bacterial classes found in giant panda fecal samples.

**Figure 2:**
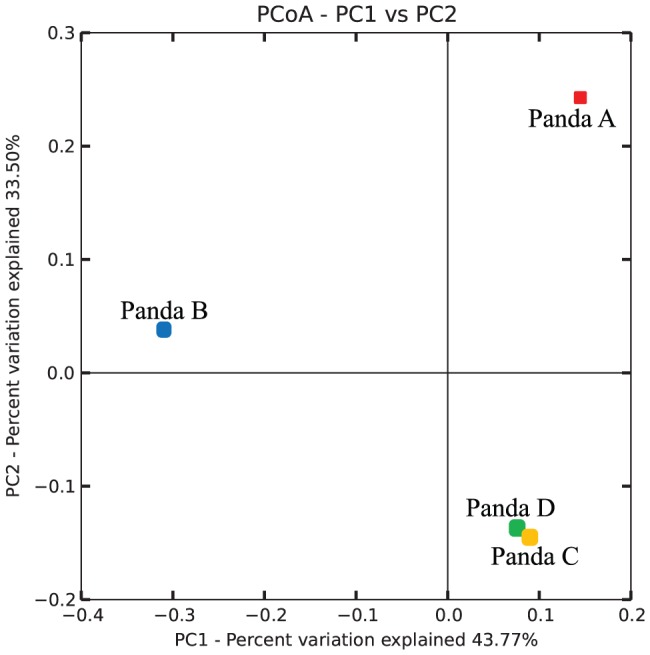
16S rRNA gene sequencing revealed a developmental change in bacterial communities in giant panda GITs. Bacterial communities were clustered using PCoA of unweighted UniFrac distance matrices. The percentages of variation shown by the plotted principal coordinates are indicated on the axes.

**Figure 3:**
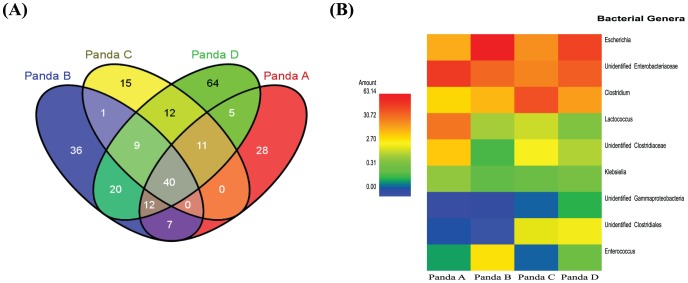
Core bacterial operational taxonomic units (OTUs) in giant panda GITs. The core community was considered based on the OTUs detected in every fecal sample. The OTUs were assigned at 97% sequences similarity threshold. (**A**) Venn diagram summarizing the numbers of common OTUs among four giant pandas. (**B**) The relative abundance of core genera found in all giant pandas. The heat map indicates the relative percentage of each bacterial genus within each panda fecal sample.

After removal of chimeric sequences, chloroplast sequences (748 sequences), and quality trimming, 93,077 good quality sequences (14,500 reads for male geriatric panda A, 29,726 reads for female geriatric panda B, 7,836 reads for male adult panda C, and 41,015 reads for female adult panda D) were retained for downstream analysis. The sequences were assigned to 259 operational taxonomic units (OTUs) at 97% threshold level. The numbers of OTUs distributed in each sample were as follows: 103 OTUs for panda A, 125 OTUs for panda B, 88 OTUs for panda C, and 173 OTUs for panda D. Both Chao1 and ACE species richness indices at the same sequencing depth for each animal showed that adult pandas (pandas C and D) bore more species richness, whereas the geriatric panda B expressed the lowest richness (Table 1). Additionally, Simpson and Shannon indices of diversity showed that panda B had the lowest bacterial diversity (Table 1). According to the average species richness indices, our sequencing effort achieved 45–73% identification of the total bacterial community from the gut of giant pandas, while the rarefaction curves did not reach saturation at 97% pairwise identity thresholds (Figure S1). At the phylum level, the most abundant bacteria were members of Firmicutes (114 OTUs, 42–79% of total sequences) and Proteobacteria (115 OTUs, 21–58% of total sequences). The relative abundance of these taxonomic phyla varied among each panda. The remainders belonged to members of Actinobacteria (0.02–0.06%), Bacteroidetes (0.002%), and to unidentified bacterial phyla (0.02–0.15%) (Figure 1). At least three taxonomic groups (Firmicutes, Proteobacteira and other unidentified phyla) were found in all four giant pandas. However, Actinobacteria was observed only in the two adult pandas C and D, while members of Bacteroidetes were present only in panda D. Among the members of Firmicutes, *Clostridiaceae* was the most abundant family member among pandas B, C, and D, while *Streptococcaceae* had the highest abundance in panda A. *Enterobacteriaceae* was the most abundant family member of phylum *Proteobacteria* in all four pandas (Table S1). At the genus level, three genera including *Actinomyces*, *Microbacterium* and *Aeromonas* were found only in adult pandas, but not in geriatric pandas (Table S1). Un-weighted UniFrac analysis delineated that the two adult pandas C and D had similar bacterial members in their gut by clustering closely on the two-dimensional PCoA plot (Figure 2), indicating possible developmental changes in the gut bacterial community among different age groups. Although different bacterial communities are harbored in the guts of pandas of different age groups, the core bacterial members of panda GITs remain the most interesting due to their common roles in the digestive physiology of the species. Among the four pandas, only 40 OTUs were identified as constituting core bacterial OTUs (Figure 3A). Upon further examination of these 40 core OTUs, the majority were identified as family *Enterobacteriaceae*, followed by *Clostridiaceae*, *Streptococcaceae* and *Enterococcaceae*. Besides the known families of bacteria, minor abundances of unidentified *Clostrida* and *Gammaproteobacteria* were identified as well (Figure 3B).

**Table 1:** Richness and diversity estimation for both bacterial and fungal diversities based on pyrotag sequence analysis.

Microbiota	Sample	Species richness indices		Species diversity indices	
		Chao1	ACE	Shannon	Simpson
Bacterial	Panda A	115	121	2.99	0.76
	Panda B	116	116	2.27	0.67
	Panda C	155	165	2.66	0.78
	Panda D	157	142	2.75	0.76
Fungal	Panda A	111	111	2.65	0.82
	Panda B	108	108	1.42	0.45
	Panda C	157	157	3.5	0.93
	Panda D	229	229	3.5	0.93

### Fungal diversity in the guts of giant pandas

A total of 26,449 ITS-1 sequences (mean length, 247 bases) passed the various quality control steps, and the numbers of reads per sample ranged from 3,273 to 10,855. The resultant sequences were subsequently clustered into OTUs at 97% similarity level. Upon removal of singletons and non-fungal sequences, the average number of OTUs detected per sample was 151 with a range of 108–229 OTUs. For each sample, the rarefaction curves trended towards saturation at 97% pairwise identity thresholds (Figure S2). Diversity indices revealed a greater fungal community variance among the two adult pandas C and D than in the geriatric pandas A and B. Analogous to our bacterial community results, the eldest panda B had the lowest fugal species richness and diversity, suggesting the effects of aging on panda GIT microbial community diversification (Table 1).

**Figure 4:**
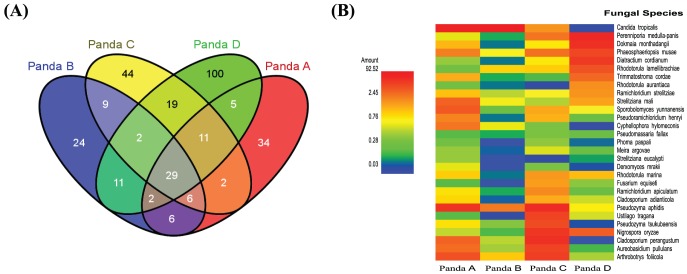
Core fungal species in giant panda GITs. The core community was considered based on the common fungal species found in every fecal sample. (**A**) Venn diagram summarizing the numbers of common fungal species among four giant pandas in this study. (**B**) The relative abundance of core fungal species found in all giant pandas. Heat map indicating the relative percentage of each fungal species within each panda fecal sample.

Most fungal OTUs identified in giant panda's GIT were affiliated with the phyla Ascomycota or Basidiomycota, except for a scarcity of reads which were identified as an early diverging fungal lineage (Table 2). The relative abundances of both phyla almost equally contributed to the microbiota of three pandas, while in the case of panda B, Ascomycota had the highest contribution. At the class level, the most abundant member of Ascomycota in the two geriatric pandas was *Saccharomycetes*, whereas *Sordariomycetes* was most abundant in the two adult pandas. However, no significant age-related differences in the Basidocmycota members in giant pandas were observed (Table 2). At the species level identification, 95 species in panda A, 89 species in panda B, 122 species in panda C, and 179 species in panda D were found. Among all the fungal species identified, only 29 species were found to be the core fungal community among the four pandas in this study (Figure 4A). The majority of core fungal species were observed to be members of the *Ascomycota* family (Figure 4B). At the species level of the core fungal community, high abundances of *Candida tropicalis* were found in both geriatric pandas, but not in the two adult pandas. Nevertheless, the most abundant core fungal species in the two adult pandas were different; *Pseudozyma aphids* in panda C and *Perenniporia medulla panis* in panda D (Table S2).

**Table 2:** Summary of the abundance and distribution of operation taxonomic units (OTUs) among fungal lineages in giant panda GITs.

Taxonomic affinity	% of total sequence reads in each panda			
	Panda A	Panda B	Panda C	Panda D
**Ascomycota**	**50.4**	**91.2**	**55.1**	**60.2**
Ascomycota incertae sedis	0.4	0.1	0.3	7.3
Mitosporic Ascomycota	-	0.1	-	0.5
Pezizomycotina				
Dothideomycetes	12.5	3.6	29.8	14.5
Eurotiomycetes	3.6	0.8	0.9	0.9
Lecanoromycetes	0.1	0.1	0.1	-
Leotiomycetes	0.2	1.1	0.1	0.1
Orbiliomycetes	1.5	0.7	4.9	0.2
Pezizomycetes	-	-	-	0.1
Sordariomycetes	1.1	0.7	17.9	36.3
Saccharomycotina				
Saccharomycetes	31	84	1	0.1
Taphrinomycotina				
Taphrinomycetes	-	-	-	0.2
**Basidomycota**	**49.5**	**8.8**	**44.8**	**39.8**
Basidomycota incertae sedis				
Wallemiomycetes	-	0.1	-	-
Agaricomycotina				
Agaricomycetes	0.9	0.3	1.7	32
Tremellomycetes	6.1	4.9	6.4	0.5
Pucciniomycotina				
Agaricostilbomycetes	-	-	0.1	0.1
Cystobasidiomycetes	4.2	1.2	2.2	5.9
Microbotryomycetes	-	0.1	-	-
Pucciniomycetes	0.1	-	-	-
Ustilaginomycotina				
Exobasidomycetes	0.1	0.2	0.4	0.2
Ustilaginomycetes	38.1	2	34	1.1
**Fungi incertae sedis (early diverging fungal lineage)**	**0.1**	-	**0.1**	-
Mortierellomycotina	0.1	-	0.1	-

### Diversity of FTHFS gene in the gut of giant pandas

**Figure 5:**
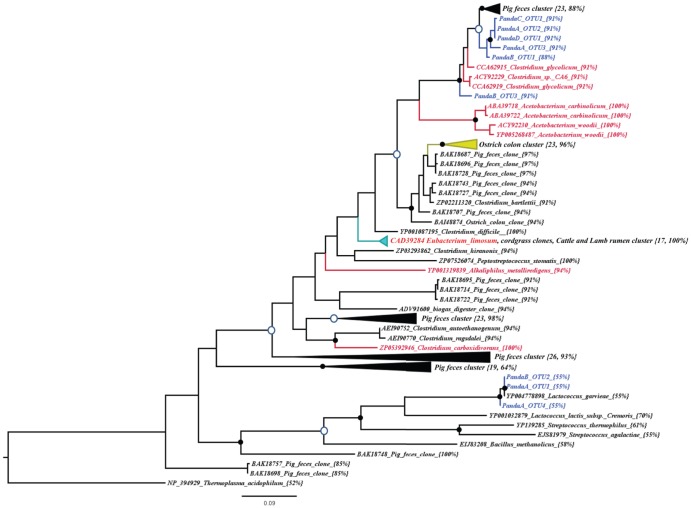
Phylogenetic tree based on 152 putative formyltetrahydrofolate synthetase (FTHFS) genes deposited in the GenBank database together with representative FTHFS genes recovered from the fecal samples of giant pandas. GenBank accession numbers of the reference sequences are shown followed by the species names. Bootstrap values of ≥70% are shown at nodes as closed circles for both tree construction methods and open circles for maximum likelihood method only. HS scores are included in parentheses for OTUs recovered in the present study. For the clusters, the number of sequences and the HS_c_ score are indicated as follows; {n, HS_c_ score}. The scale bar shows 9% sequence divergence.

A total of 50 FTHFS sequences for each giant panda were subjected to RFLP analysis and sequencing. After HinP1I digestion, three RFLP patterns were found from the sequences libraries of geriatric pandas. However, only one RFLP pattern was found among the cloned sequences from the adult pandas. At 98% amino acid similarity level, 3–4 OTUs were assigned for the cloned sequences from pandas A and B, while only one OTU was found among the cloned sequences of pandas C and D. The BLAST matches indicated that most panda OTUs had a maximum of 96% protein similarity to the putative FTHFS sequences from uncultured organisms originated from pig feces, except for three OTUs (PandaA-OTU1, 4 and PandaB-OTU2), that were strongly matched to the FTHFS gene of cultured *Lactococcus garvieae*, a fish pathogen. Our panda-derived FTHFS sequences were unrelated to the FTHFS sequences from plants, suggesting that the panda FTHFS sequences are unlikely to be of plant origin. Both neighbor joining (NJ) and maximum likelihood (ML) trees resulted in similar tree topologies. From our phylogenic analysis, the PandaA-OTU1, 4 and PandaB-OTU2 with low HS score (55%) were closely related to the FTHFS sequence from a cultured non-homoacetogen, *Lactococcus garvieae*. However, other panda OTUs with high HS scores (88–91%) formed novel phylotypes with high bootstrap supports (Figure 5).

## Discussion

As an iconic species for global conservation, the giant panda is threatened by a number of adversities which keep population numbers very low [29]. Due to anthropogenic disturbances such as habitat destruction and land partitioning that results in fragmented nonviable populations, scientific research on the giant panda is needed to support the effective conservation of the species [29]. In the management of giant pandas at zoological institutions, nutritional considerations play a major role since digestive pathologies are over-represented among all age classes, often in the form of chronic conditions [24]. Moreover, the unique physical and behavioral discord among giant pandas as a herbivorous carnivore imposes intriguing research questions with regards to its evolutionary history, whereby dietary adaptation is a major driving force for the evolution of all species [30]. As in other microbiome projects, our major goal is to develop effective strategies for the manipulation of gut microbial communities to promote overall panda health. Hence, an in-depth knowledge of the bacterial diversity and the principles governing microbial community assembly are the first steps needed to achieve our long-term goals. Previous research employed relatively shallow sampling to detect the bacterial diversity in the gut of giant pandas [7]–[10], and none of these studies had previously assessed fungal diversity. Moreover, the core gut microbial community, as well as composition differences between adult and geriatric pandas remains unidentified. These limitations have been overcome to a certain extent in this study by our sampling and sequencing efforts.

The rarefaction and species richness indices for bacterial diversity indicated that further deep sequencing is needed to achieve a comprehensive understanding of bacterial diversity in the panda GIT. Although our sampling efforts did not reveal the entire bacterial community, more than 100 OTUs were identified for each panda. These results contradicted with the previous findings, which reported lower species richness (<100 OTUs) in the giant panda GIT. Likewise, the Shannon index for bacterial diversity in this study (2.3–3.0) was higher than that of previous findings [9]. The fundamental differences in sequencing depth, as well as the possible physiological and environmental differences between the pandas in our current study and those in other previous studies provide the best explanation to these contradictory findings. It is also worthwhile to note that the saturation of rarefaction curves and fungal diversity indices indicated an effective description of the entire fungal community in our study. To our knowledge, this is the first endeavor to explore the entire fungal community in the giant panda GIT. These findings suggest that anaerobic fungi, much like other cellulolytic bacteria, may have a potential role in degrading plant materials. Therefore, the functional ecology between bacteria and fungi in the panda GIT needs further investigations.

From the comparison of diversity indices among the four pandas, the eldest female panda (B) showed the lowest bacterial species richness and diversity. Among the human elderly, the composition of the intestinal microbiota changes due to decreased species diversity and this may result in reduced levels of beneficial bacteria [27], [31]. Such microbial composition changes have been explained by alterations in intestinal mobility and nutrient availability that occur with aging [31]. In addition, psychosocial stress factors can also contribute to changes in the immune system that may affect the composition of the human gut microbiota [32]. Among the observed bacterial phyla, Actinobacteria was absent from both geriatric pandas. Among the human elderly, the reduction in a member of Actinobacteria (*Bifidobacterium* species) has been previously reported [33]. However, no *Bifidobacteria* species were observed in any panda from this study. It is formally possible that *Bifidobacteria* were under-represented here because either the general primers utilized in this study failed to amplify, and/or the low abundance of this genus in the samples was consistently inadequate for detection, thus necessitating further investigation. Actinobacteria are common in natural environments such as soil, fresh and marine water, and termites. In termites, Actinobacteria function as defensive endosymbionts [34]. Furthermore, several members of phylum Actinobacteria were already known to have the capability to produce enzymes that degrade plant organic compounds such as cellulose and chitin [35]–[37]. However, the functional roles of cellulolytic Actinobacteria have yet to be identified. At the genus level, only two genera (*Actinomyces* and *Microbacterium*) of Actinobacteria are common to both adult pandas, while other genera are present only in one. Both *Actinomyces* and *Microbacterium* have been reported previously to be cellulose decomposers [36]–[38]. Therefore, further investigation should be done to study the roles of these genera in cellulose and lignin metabolism in the giant panda GIT, as well as the impact of their absence in geriatric pandas. In contrast to the human elderly, no significant variation of enterobacteria composition was observed in the guts of both geriatric and adult pandas.

To the authors' knowledge, this is the first study to explore the fungal community in the giant panda GIT. Only two fungal phyla (Ascomycota and Basidomycota) were found in panda guts. Similar to our bacterial diversity results, panda B had the least fungal diversity and species richness. *Candida tropicalis* was found in higher abundance in the GIT of geriatric pandas. *Candida tropicalis* do not only colonize the GITs of animals, but also exert nutritional and other probiotic effects on the hosts [39]. Moreover, this species of fungus has been reported to be a virulent fungal pathogen [40]. A white-rot fungus, *Perenniporia medulla-panis*, was identified in all four giant pandas, with the highest abundance in the adult panda D. *Perenniporia medulla-panis* has been known as a lignocellulolytic basidomycete, which produces an extracellular enzyme (lignin peroxidase) essential for lignin degradation [41]. The digestion of lignin and lignin-related compounds found in bamboo in the giant panda GIT has been recently studied by screening for key lignin degrading enzymes (laccases) [10]. In nature, white-rot fungi are the major microorganisms for degrading lignocellulose compounds [41]. Yet, no study has shown the roles of fungal lignocelluloytic pathways in the giant panda metabolism. Our fungal diversity data highlights the need for further studies on the fungi colonizing giant panda GITs and their roles in bamboo digestion.

Core microbial communities were determined in every individual panda. Only 40 bacterial OTUs and 29 fungal species were found in common to all four pandas. As this study was conducted with four animals, increasing the sample size will yield additional data that will further improve these estimations, resulting in higher confidence levels. In addition, performing deeper sampling for bacterial diversity will aid in identifying less abundant core communities.

In addition to determining the overall composition of bacterial and fungal communities, we discovered the diversity of homoacetogenic bacteria (acetogens) by clone library sequencing using a primer set to amplify partial (1,102 of 1,680 bp) FTHFS gene sequences. Acetogens utilize H_2_ to reduce CO_2_ and form a volatile fatty acid (Acetate), which can be used as an energy source by giant pandas. The number of observed FTHFS OTUs in geriatric pandas is higher than that in adult pandas. The diversity of FTHFS gene fragments in giant pandas is significantly lower than that in other vertebrates [15]–[17]. The separation of panda FTHFS OTUs from the pig feces cluster was well supported in both treeing methods. These novel uncultured FTHFS phylotypes indicated the presence of as-yet-unknown homoacetogens in giant panda GITs. Meanwhile, other unique OTUs from geriatric pandas possessed few of the amino acid residues characteristic of FTHFS from known homoaceotgens and did not cluster with those from the known homoacetogens. Thus, these FTHFS-like OTUs may be xenologs, analogs or homologs of the FTHFS gene. FTHFS could also be used in the metabolism of some sulfate-reducing bacteria [16]. However, none of the panda-derived FTHFS sequences clustered with those of sulfate reducers or *Treponema* spp. from termites.

In conclusion, this is the first study to characterize both bacterial and fungal communities concurrently in both geriatric and adult giant pandas using 16S pyrotag sequencing. We also identified the presence of novel homoacetogens in the guts of giant pandas. Further investigation focusing on the functional characteristics of the microbiota is critically important for understanding the composition and activity of the intestinal microbiota associated with ageing in giant pandas. Furthermore, isolation of both beneficial prokaryotes and eukaryotes may prove to be an optimal platform for the development of probiotics specific to giant panda dietary requirements and to their overall health.

## Materials and Methods

### Ethics statement

This study was carried out with zoological institution approval (Ocean Park Corporation) in Hong Kong. The sampling was performed by the curators according to protocols approved by the zoological institution.

### Giant panda husbandry

All samples were obtained in accordance to ethical guidelines. All four pandas (geriatric; n = 2 and adult; n = 2) in this study are housed at a zoological institution in Hong Kong, providing an environment reminiscent of their natural habitat. Notably, one of the two geriatric pandas (37 years old, Panda B) is the oldest female giant panda in the world. Table S3 provides details of the four animals characterized in this study. Bamboo was the major component of their diet with their preferred species of bamboo provided *ad libitum*. Table S4 shows the bamboo species selected by individual pandas. Each giant panda daily consumed an average of 7 kilograms of bamboo, representing approximately half of the amount provided. Besides bamboos, 1–2 kilograms of additional supplementary foods such as vegetables and fruits (e.g. carrots, apples, pears etc.) and high fibrous biscuits were also provided to the pandas.

### Sample collection, preparation and DNA extraction

At the time of sampling, all four pandas were free of any digestive symptoms and produced normal feces (grade 2 fecal materials according to the fecal grading system, Edwards et al, 2006). Fresh fecal samples were collected in sterile plastic bags, rapidly stored at -40^o^C, and delivered to the laboratory on dry ice. Upon arrival, fecal materials were chilled by liquid nitrogen and grinded by mortar and pestle. Two DNA extractions were performed for fecal homogenates from each panda (technical replicates) with the PowerSoil DNA Isolation Kit (Carlsbad, CA, USA) and a method modified by Tun et al, 2012 [42]. Extracted DNA was quantified by a Nanodrop 2000 spectrophotometer (Thermo Scientific, Wilmington, DE, USA) and stored at −80°C until subsequent procedures.

### PCR amplification and pyrosequencing

PCR amplifications were performed using the FastStart High Fidelity PCR System (Roche Molecular Diagnostics, Branchburg, NJ, USA). For bacterial diversity, the primer pair 530F (5′-GTGCCAGCMGCNGCGG) and 1100R (5′- GGGTTNCGNTCGTTG) was used to amplify a ∼600 bp fragment from the V4–6 hypervariable regions of 16S rRNA gene. For fungal diversity, the primers ITS1F (5′- CTTGGTCATTTAGAGGAAGTAA) and ITS2 (5′-GCTGCGTTCTTCATCGATGC) were used to generate ∼300–400 bp of the variable ITS-1 regions. Four sets of 9 nucleotides barcode were designed by “Barcrawl” [43] and incorporated into the 5′ends of forward primers for multiplex pyrotag sequencing. Amplicon libraries were subjected to sequencing using the 454 GS Junior System (454 Life Sciences-a Roche Company, Branford, CT, USA). Tag-encoded pyrosequence data were deposited into NCBI Sequence Read Archive under accession numbers; SRA052360.1 for bacterial and SRA064952 for fungal data.

### Data analysis

For bacterial diversity analysis, 16S rDNA sequences generated from 454 GS Junior sequencer were processed by the QIIME (quantitative insights into microbial ecology) pipeline [44]. Briefly, sequences with mean quality score lower than 25, <200 bp or >1,000 bp in lengths, incorrect primer sequences, or more than 1 ambiguous base were discarded. The sequences were de-multiplexed based on their respective barcode sequences. Denoising of the pyrotag sequences was performed using DENOISER v. 0.9.1 [45] as implemented in the QIIME platform. Chimeric sequences were removed using ChimeraSlayer. Sequences were clustered into Operational Taxonomic Units (OTUs) at the threshold of 97% sequence similarity. β-diversity analysis generated a principal coordinate plot using un-weighted UniFrac distance implemented in QIIME [44]. A Venn diagram was generated using custom Perl scripts to identify the number of core OTUs among the four giant pandas.

For fungal diversity analysis, generated ITS1 sequences shorter than 140 bp after quality trimming were excluded from analysis. The Fungal ITS Extractor 1.1 [46] was applied to extract the variable ITS1 subregion of fungal ITS sequences and to exclude any portions of neighboring ribosomal genes. Raw sequences without pyrotags and forward primer sequences, as well as non-fungal ITS sequences were excluded from the analysis. Removal of ITS chimeric sequences was performed using a blast-based open source software package (available at http://www.emerencia.org/chimerachecker.html). The resultant sequences for individual samples were subjected to the pyrosequencing analysis pipeline at UNITE database [47]. The rarefaction analysis for both bacterial and fungal data was performed using the ANALYTIC RAREFACTION v.1.4 (Hunt Mountain Software, Department of Geology, University of Georgia, Athens, GA, USA) and the calculation of Shannon richness and Chao1 diversity indices was performed using the ESTIMATES v.8.0 [48]. Alpha-diversity indices were calculated based on the 7,800 bacterial 16S rDNA reads and 3,200 fungal ITS1 reads analyzed from each panda. The core fungal community was analysed based on the fungal species present in individual pandas.

### Clone library construction for FTHFS genes and sequence analysis

FTHFS genes were amplified from the extracted panda fecal genomic DNA as described by Leaphart and Lovell [49]. PCR products were purified using PCR purification kit and cloned using a pGEM-T Easy vector system (Promega, Madison, WI, USA). Subsequently, 50 positively cloned PCR products were screened by restriction fragment length polymorphism (RFLP) analysis using HinP1I as described by Ottesen and Leadbetter [18]. Regardless of the RFLP patterns, 50 positive clones from each sample were subjected for Sanger sequencing using the vector primers (M13F and M13R). Sequences were assembled and edited using the Lasergene software package v.7.2.1 [50]. FTHFS protein sequences were aligned using MEGA5 software [51], and checked for chimeric sequences in the Bellerophon program (no chimeric sequence were found) [52]. The aligned protein sequences were clustered into Operational Taxonomic Units (OTUs) at the threshold of 98% similarity using UCLUST [53]. Phylogenetic analyses of giant panda FTHFS clones were performed using 317 unambiguous, aligned amino acids, and both neighbor-joining and maximum likelihood trees were constructed using PhyML v.3 [54] and MEGA5 [51]. Homoacetogen similarity (HS) scores were calculated for panda FTHFS clones. The sequences were deposited in GenBank under accession numbers KC424783-KC424981.

## Supporting Information

Figure S1 Rarefaction curves of bacterial OTUs identified from four giant pandas, clustered at 97% sequence identity.
(EPS)

Figure S2 Rarefaction curves of fungal OTUs identified from four giant pandas, clustered at 97% sequence identity.
(EPS)

Table S1 Summary of the relative distribution of different bacteria genera in the guts of giant pandas.
(DOC)

Table S2 Summary of the relative distribution of fungi (subclass level) in the guts of giant pandas.
(DOC)

Table S3 Details of the four animals involved in the study.
(DOC)

Table S4 List of bamboo species preferred by individual giant pandas.
(DOC)
