# Supplementary material for: Microbial Diversity and Evidence of Novel Homoacetogens in the Gut of Both Geriatric and Adult Giant Pandas (Ailuropoda melanoleuca)
Source: PLoS One. 2014 Jan 24;9(1):e79902. doi: 10.1371/journal.pone.0079902 (PMC3901650; doi:10.1371/journal.pone.0079902)
Supplement: Table S1 — Summary of the relative distribution of different bacteria genera in the guts of giant pandas. (DOC) [file pone.0079902.s003.doc]

**Supplementary Table 1. Summary of the relative distribution of different bacteria genera in the guts of** giant pandas.

| **Phylum** | **Class** | **Order** | **Family** | **Genus** | **Panda A** | **Panda B** | **Panda C** | **Panda D** |
| --- | --- | --- | --- | --- | --- | --- | --- | --- |
| Unknown Bacteria | Other | Other | Other | Other | 0.12 | 0.10 | 0.05 | 0.02 |
| Actinobacteria | Actinobacteria | Actinomycetales | Actinomycetaceae | Actinomyces | 0.00 | 0.00 | 0.01 | 0.00 |
| Actinobacteria | Actinobacteria | Actinomycetales | Actinomycetaceae | Arcanobacterium | 0.00 | 0.00 | 0.00 | 0.00 |
| Actinobacteria | Actinobacteria | Actinomycetales | Corynebacteriaceae | Corynebacterium | 0.00 | 0.00 | 0.00 | 0.00 |
| Actinobacteria | Actinobacteria | Actinomycetales | Microbacteriaceae | Curtobacterium | 0.00 | 0.00 | 0.00 | 0.00 |
| Actinobacteria | Actinobacteria | Actinomycetales | Microbacteriaceae | Microbacterium | 0.00 | 0.00 | 0.03 | 0.00 |
| Actinobacteria | Actinobacteria | Actinomycetales | Propionibacteriaceae | Propionibacterium | 0.00 | 0.00 | 0.01 | 0.00 |
| Actinobacteria | Actinobacteria | Solirubrobacterales | Patulibacteraceae | Patulibacter | 0.00 | 0.00 | 0.01 | 0.00 |
| Bacteroidetes | Flavobacteria | Flavobacteriales | Flavobacteriaceae | Other | 0.00 | 0.00 | 0.00 | 0.00 |
| Firmicutes | Other | Other | Other | Other | 0.02 | 0.00 | 0.00 | 0.00 |
| Firmicutes | Bacilli | Bacillales | Other | Other | 0.00 | 0.00 | 0.00 | 0.00 |
| Firmicutes | Bacilli | Lactobacillales | Other | Other | 0.02 | 0.00 | 0.00 | 0.01 |
| Firmicutes | Bacilli | Lactobacillales | Enterococcaceae | Other | 0.00 | 0.01 | 0.00 | 0.01 |
| Firmicutes | Bacilli | Lactobacillales | Enterococcaceae | Enterococcus | 0.05 | 1.35 | 0.03 | 0.13 |
| Firmicutes | Bacilli | Lactobacillales | Lactobacillaceae | Lactobacillus | 0.00 | 0.02 | 0.00 | 0.00 |
| Firmicutes | Bacilli | Lactobacillales | Lactobacillaceae | Pediococcus | 0.00 | 0.00 | 0.00 | 0.00 |
| Firmicutes | Bacilli | Lactobacillales | Leuconostocaceae | Leuconostoc | 6.39 | 0.24 | 0.00 | 0.01 |
| Firmicutes | Bacilli | Lactobacillales | Leuconostocaceae | Weissella | 3.77 | 0.99 | 0.00 | 0.41 |
| Firmicutes | Bacilli | Lactobacillales | Streptococcaceae | Lactococcus | 23.03 | 0.26 | 0.42 | 0.18 |
| Firmicutes | Bacilli | Lactobacillales | Streptococcaceae | Streptococcus | 1.30 | 0.01 | 0.01 | 0.80 |
| Firmicutes | Bacilli | Turicibacterales | Turicibacteraceae | Other | 0.03 | 0.00 | 1.16 | 2.14 |
| Firmicutes | Bacilli | Turicibacterales | Turicibacteraceae | Turicibacter | 0.01 | 0.00 | 0.13 | 0.19 |
| Firmicutes | Clostridia | Clostridiales | Other | Other | 0.01 | 0.01 | 0.52 | 0.79 |
| Firmicutes | Clostridia | Clostridiales | Clostridiaceae | Other | 0.31 | 0.08 | 1.38 | 0.88 |
| Firmicutes | Clostridia | Clostridiales | Clostridiaceae |  | 6.31 | 0.00 | 18.64 | 10.21 |
| Firmicutes | Clostridia | Clostridiales | Clostridiaceae | Clostridium | 2.93 | 4.73 | 35.23 | 14.61 |
| Firmicutes | Clostridia | Clostridiales | Clostridiaceae | Sarcina | 0.81 | 13.59 | 0.00 | 0.00 |
| Firmicutes | Clostridia | Clostridiales | ClostridialesFamilyXI.IncertaeSedis | Other | 0.00 | 0.00 | 0.01 | 0.00 |
| Firmicutes | Clostridia | Clostridiales | Lachnospiraceae | Other | 0.00 | 0.00 | 0.00 | 0.00 |
| Firmicutes | Clostridia | Clostridiales | Lachnospiraceae | Epulopiscium | 0.01 | 0.00 | 0.00 | 0.00 |
| Firmicutes | Clostridia | Clostridiales | Peptostreptococcaceae | Other | 0.00 | 0.00 | 0.00 | 0.03 |
| Firmicutes | Clostridia | Clostridiales | Peptostreptococcaceae | Tepidibacter | 0.00 | 0.00 | 0.00 | 0.00 |
| Firmicutes | Clostridia | Clostridiales | Veillonellaceae | Veillonella | 0.01 | 0.00 | 0.00 | 0.00 |
| Proteobacteria | Other | Other | Other | Other | 0.00 | 0.00 | 0.00 | 0.00 |
| Proteobacteria | Alphaproteobacteria | Rhizobiales | Other | Other | 0.00 | 0.00 | 0.00 | 0.00 |
| Proteobacteria | Alphaproteobacteria | Rhizobiales | Methylobacteriaceae | Methylobacterium | 0.00 | 0.00 | 0.00 | 0.01 |
| Proteobacteria | Alphaproteobacteria | Rhizobiales | Rhizobiaceae | Agrobacterium | 0.00 | 0.00 | 0.00 | 0.00 |
| Proteobacteria | Alphaproteobacteria | Sphingomonadales | Sphingomonadaceae | Sphingomonas | 0.00 | 0.00 | 0.00 | 0.00 |
| Proteobacteria | Epsilonproteobacteria | Campylobacterales | Campylobacteraceae | Campylobacter | 0.00 | 0.54 | 0.05 | 0.14 |
| Proteobacteria | Epsilonproteobacteria | Campylobacterales | Helicobacteraceae | Other | 0.00 | 0.00 | 0.00 | 0.01 |
| Proteobacteria | Epsilonproteobacteria | Campylobacterales | Helicobacteraceae | Helicobacter | 0.00 | 0.00 | 0.00 | 0.56 |
| Proteobacteria | Gammaproteobacteria | Other | Other | Other | 0.01 | 0.01 | 0.03 | 0.06 |
| Proteobacteria | Gammaproteobacteria | Aeromonadales | Aeromonadaceae | Aeromonas | 0.00 | 0.00 | 0.04 | 0.03 |
| Proteobacteria | Gammaproteobacteria | Enterobacteriales | Enterobacteriaceae | Other | 49.64 | 25.67 | 21.61 | 28.76 |
| Proteobacteria | Gammaproteobacteria | Enterobacteriales | Enterobacteriaceae | Cedecea | 0.00 | 0.00 | 0.00 | 0.00 |
| Proteobacteria | Gammaproteobacteria | Enterobacteriales | Enterobacteriaceae | Escherichia | 4.90 | 52.22 | 20.43 | 39.62 |
| Proteobacteria | Gammaproteobacteria | Enterobacteriales | Enterobacteriaceae | Klebsiella | 0.24 | 0.12 | 0.11 | 0.15 |
| Proteobacteria | Gammaproteobacteria | Enterobacteriales | Enterobacteriaceae | Morganella | 0.06 | 0.00 | 0.05 | 0.17 |
| Proteobacteria | Gammaproteobacteria | Enterobacteriales | Enterobacteriaceae | Salmonella | 0.01 | 0.01 | 0.00 | 0.02 |
| Proteobacteria | Gammaproteobacteria | Enterobacteriales | Enterobacteriaceae | Trabulsiella | 0.00 | 0.01 | 0.00 | 0.00 |
| Proteobacteria | Gammaproteobacteria | Pasteurellales | Pasteurellaceae | Other | 0.00 | 0.00 | 0.00 | 0.02 |
| Proteobacteria | Gammaproteobacteria | Pseudomonadales | Moraxellaceae |  | 0.00 | 0.00 | 0.01 | 0.00 |
| Proteobacteria | Gammaproteobacteria | Pseudomonadales | Moraxellaceae | Acinetobacter | 0.00 | 0.00 | 0.01 | 0.00 |
| Proteobacteria | Gammaproteobacteria | Pseudomonadales | Moraxellaceae | Psychrobacter | 0.01 | 0.00 | 0.00 | 0.00 |
