# Supplementary material for: Microbial Diversity and Evidence of Novel Homoacetogens in the Gut of Both Geriatric and Adult Giant Pandas (Ailuropoda melanoleuca)
Source: PLoS One. 2014 Jan 24;9(1):e79902. doi: 10.1371/journal.pone.0079902 (PMC3901650; doi:10.1371/journal.pone.0079902)
Supplement: Table S2 — Summary of the relative distribution of fungi (subclass level) in the guts of giant pandas. (DOC) [file pone.0079902.s004.doc]

**Supplementary Table 2. Summary of the relative distribution of fungi (subclass level) in the guts** of giant pandas.

| **Division/Phylum** | **Subdivision** | **Class** | **Subclass** | **Panda A** | **Panda B** | **Panda C** | **Panda D** |
| --- | --- | --- | --- | --- | --- | --- | --- |
| Ascomycota |  |  |  |  |  |  |  |
|  | Ascomycota incertae sedis |  |  |  |  |  |  |
|  |  |  | Aporospora | 0.00 | 0.00 | 0.02 | 0.00 |
|  |  |  | Dokmaia. | 0.46 | 0.07 | 7.29 | 0.34 |
|  | mitosporic Ascomycota |  |  |  |  |  |  |
|  |  |  | Acremonium | 0.00 | 0.00 | 0.11 | 0.00 |
|  |  |  | Acrodontium | 0.00 | 0.04 | 0.03 | 0.00 |
|  |  |  | Flagellospora. | 0.00 | 0.04 | 0.00 | 0.00 |
|  |  |  | Humicola | 0.00 | 0.00 | 0.02 | 0.00 |
|  |  |  | Microsphaeropsis | 0.00 | 0.00 | 0.06 | 0.00 |
|  |  |  | Ochroconis | 0.00 | 0.00 | 0.21 | 0.00 |
|  |  |  | Phialemonium | 0.00 | 0.00 | 0.02 | 0.00 |
|  |  |  | Pseudodictyosporium | 0.00 | 0.00 | 0.09 | 0.00 |
|  |  |  |  |  |  |  |  |
|  | Pezizomycotina | Dothideomycetes |  |  |  |  |  |
|  |  |  | Dothideomycetidae | 10.27 | 2.96 | 4.29 | 25.46 |
|  |  |  | Unclassified Dothideomycetes (Eppicocum) | 2.05 | 0.06 | 0.02 | 0.17 |
|  |  |  | Dothideomycetes incertae sedis | 0.00 | 0.00 | 0.04 | 0.00 |
|  |  |  | Pleosporomycetidae | 0.18 | 0.59 | 10.12 | 4.22 |
|  |  | Eurotiomycetes |  |  |  |  |  |
|  |  |  | Chaetothyriomycetidae | 2.38 | 0.73 | 0.93 | 0.60 |
|  |  |  | Eurotiomycetidae | 1.22 | 0.04 | 0.04 | 0.30 |
|  |  | Lecanoromycetes |  |  |  |  |  |
|  |  |  | Lecanoromycetidae | 0.06 | 0.03 | 0.00 | 0.00 |
|  |  |  | Lecanoromycetes incertae sedis | 0.00 | 0.00 | 0.00 | 0.09 |
|  |  |  | Ostropomycetidae | 0.06 | 0.00 | 0.00 | 0.00 |
|  |  | Leotiomycetes |  |  |  |  |  |
|  |  |  | Erysiphales | 0.06 | 0.00 | 0.00 | 0.00 |
|  |  |  | Helotiales | 0.09 | 0.00 | 0.05 | 0.04 |
|  |  |  | Leotiomycetes incertae sedis | 0.00 | 1.16 | 0.00 | 0.00 |
|  |  | Orbiliomycetes |  |  |  |  |  |
|  |  |  | Orbiliales | 1.53 | 0.75 | 0.23 | 4.95 |
|  |  | Pezizomycetes |  |  |  |  |  |
|  |  |  | Pezizales | 0.00 | 0.00 | 0.05 | 0.00 |
|  |  | Sordariomycetes |  |  |  |  |  |
|  |  |  | Hypocreomycetidae | 0.70 | 0.26 | 1.57 | 2.44 |
|  |  |  | mitosporic Sordariomycetes | 0.00 | 0.03 | 0.06 | 0.00 |
|  |  |  | Sordariomycetes incertae sedis | 0.15 | 0.23 | 15.39 | 9.33 |
|  |  |  | Sordariomycetidae | 0.12 | 0.07 | 19.12 | 5.88 |
|  |  |  | Xylariomycetidae | 0.12 | 0.09 | 0.16 | 0.24 |
|  | Saccharomycotina |  |  |  |  |  |  |
|  |  | Saccharomycetes |  |  |  |  |  |
|  |  |  | Saccharomycetales | 30.95 | 84.06 | 0.12 | 1.05 |
|  | Taphrinomycotina |  |  |  |  |  |  |
|  |  | Taphrinomycetes |  |  |  |  |  |
|  |  |  | Taphrinales | 0.00 | 0.00 | 0.18 | 0.00 |
|  |  |  |  |  |  |  |  |
|  |  |  |  |  |  |  |  |
|  |  |  |  |  |  |  |  |
|  |  |  |  |  |  |  |  |
| Basidiomycota |  |  |  |  |  |  |  |
|  | Basidiomycota incertae sedis | |  |  |  |  |  |
|  |  | Wallemiomycetes |  |  |  |  |  |
|  |  |  | Wallemiales | 0.00 | 0.06 | 0.00 | 0.00 |
|  | Agaricomycotina |  |  |  |  |  |  |
|  |  | Agaricomycetes |  |  |  |  |  |
|  |  |  | Agaricomycetidae | 0.40 | 0.22 | 1.18 | 0.22 |
|  |  |  | Agaricomycetes incertae sedis | 0.00 | 0.00 | 0.03 | 0.00 |
|  |  |  | Cantharellales | 0.00 | 0.00 | 0.02 | 0.00 |
|  |  |  | Corticiales | 0.00 | 0.00 | 0.19 | 0.04 |
|  |  |  | Hymenochaetales | 0.00 | 0.00 | 0.02 | 0.00 |
|  |  |  | Phallomycetidae | 0.09 | 0.00 | 2.12 | 0.11 |
|  |  |  | Polyporales | 0.40 | 0.13 | 28.45 | 1.27 |
|  |  |  | Russulales | 0.00 | 0.00 | 0.00 | 0.13 |
|  |  | Tremellomycetes |  |  |  |  |  |
|  |  |  | Filobasidiales | 0.00 | 0.00 | 0.07 | 0.60 |
|  |  |  | Tremellales | 6.11 | 4.94 | 0.42 | 5.79 |
|  |  |  |  |  |  |  |  |
|  | Pucciniomycotina |  |  |  |  |  |  |
|  |  | Agaricostilbomycetes |  |  |  |  |  |
|  |  |  | Agaricostilbomycetes incertae sedis | 0.00 | 0.00 | 0.06 | 0.07 |
|  |  | Cystobasidiomycetes |  |  |  |  |  |
|  |  |  | Erythrobasidiales | 4.19 | 1.19 | 5.89 | 2.22 |
|  |  | Microbotryomycetes |  |  |  |  |  |
|  |  |  | Sporidiobolales | 0.00 | 0.09 | 0.00 | 0.00 |
|  |  | Pucciniomycetes |  |  |  |  |  |
|  |  |  | Septobasidiales | 0.06 | 0.00 | 0.00 | 0.00 |
|  | Ustilaginomycotina |  |  |  |  |  |  |
|  |  | Exobasidiomycetes |  |  |  |  |  |
|  |  |  | Exobasidiales | 0.00 | 0.00 | 0.09 | 0.00 |
|  |  |  | Exobasidiomycetidae incertae sedis | 0.12 | 0.06 | 0.09 | 0.32 |
|  |  |  | Malasseziales | 0.00 | 0.13 | 0.02 | 0.09 |
|  |  | Ustilaginomycetes |  |  |  |  |  |
|  |  |  | Ustilaginales | 38.16 | 1.98 | 1.15 | 34.01 |
| Fungi incertae sedis (Early diverging fungal lineages ) | | |  |  |  |  |  |
|  | Mortierellomycotina |  |  |  |  |  |  |
|  |  |  | Mortierellales; Mortierellaceae | 0.06 | 0.00 | 0.00 | 0.04 |
