# Supplementary material for: Microbial Diversity and Evidence of Novel Homoacetogens in the Gut of Both Geriatric and Adult Giant Pandas (Ailuropoda melanoleuca)
Source: PLoS One. 2014 Jan 24;9(1):e79902. doi: 10.1371/journal.pone.0079902 (PMC3901650; doi:10.1371/journal.pone.0079902)
Supplement: Table S3 — Details of the four animals involved in the study. (DOC) [file pone.0079902.s005.doc]

**Supplementary Table 3**. Details of the four animals involved in the study.

| **Animal ID** | **Sex** | **Age (years)** | **Remarks** |
| --- | --- | --- | --- |
| Giant panda A | Male | 26 (Geriatric) | Born in the wild |
| Giant panda B | Female | 34 (Geriatric) | Born in the wild |
| Giant panda C | Male | 7 (Adult) | Captive born |
| Giant panda D | Female | 7 (Adult) | Captive born |
