# Supplementary material for: Microbial Diversity and Evidence of Novel Homoacetogens in the Gut of Both Geriatric and Adult Giant Pandas (Ailuropoda melanoleuca)
Source: PLoS One. 2014 Jan 24;9(1):e79902. doi: 10.1371/journal.pone.0079902 (PMC3901650; doi:10.1371/journal.pone.0079902)
Supplement: Table S4 — List of bamboo species preferred by individual giant pandas. (DOC) [file pone.0079902.s006.doc]

**Supplementary Table 4**. List of bamboo species preferred by individual giant pandas.

| **Panda ID** | **Prefered bamboo species** |
| --- | --- |
| Giant Panda A | Dendrocalamus brandisii  Dendrocalamus latiflorus  Phyllostachys bambusoides  Phyllostachys nigra  Pseudosasa hindsii  Pseudosasa japonica |
| Giant Panda B | Bambusa vario striata  Dendrocalamus brandisii  Dendrocalamus latiflorus  Phyllostachys nigra  Physllostachys vivax  Pseudosasa hindsii  Pseudosasa japonica |
| Giant Panda C | Dendrocalamus brandisii  Dendrocalamus latiflorus  Phyllostachys bambusoides |
| Giant Panda D | Dendrocalamus brandisii  Phyllostachys bambusoides  Phyllostachys nigra  Pseudosasa japonica |
